# Automatic Estimation of the Most Likely Drug Combination in Electronic Health Records Using the Smooth Algorithm: Development and Validation Study

**DOI:** 10.2196/37976

**Published:** 2022-11-15

**Authors:** Dan Ouchi, Maria Giner-Soriano, Ainhoa Gómez-Lumbreras, Cristina Vedia Urgell, Ferran Torres, Rosa Morros

**Affiliations:** 1 Fundació Institut Universitari per a la recerca a l'Atenció Primària de Salut Jordi Gol i Gurina Barcelona Spain; 2 Facultat de Medicina. Departament de Farmacologia, Toxicologia i Terapèutica Universitat Autònoma de Barcelona Bellaterra (Cerdanyola del Vallés) Spain; 3 Unitat de Farmacia. Servei d’Atenció Primaria Barcelonès Nord i Maresme Institut Català de la Salut Barcelona Spain; 4 Department of Pharmacotherapy College of Pharmacy University of Utah Salt Lake City, UT United States; 5 Unitat de Bioestadística Facultat de Medicina Universitat Autònoma de Barcelona Bellaterra (Cerdanyola del Vallès) Spain; 6 Spanish Clinical Research Network Platform Barcelona Spain

**Keywords:** electronic health records, data mining, complex drug patterns, algorithms, drug utilization, polypharmacy, EHR, medication, drug combination, therapy, automation, drug exposition, treatment, adherence

## Abstract

**Background:**

Since the use of electronic health records (EHRs) in an automated way, pharmacovigilance or pharmacoepidemiology studies have been used to characterize the therapy using different algorithms. Although progress has been made in this area for monotherapy, with combinations of 2 or more drugs the challenge to characterize the treatment increases significantly, and more research is needed.

**Objective:**

The goal of the research was to develop and describe a novel algorithm that automatically returns the most likely therapy of one drug or combinations of 2 or more drugs over time.

**Methods:**

We used the Information System for Research in Primary Care as our reference EHR platform for the smooth algorithm development. The algorithm was inspired by statistical methods based on moving averages and depends on a parameter *Wt*, a flexible window that determines the level of smoothing. The effect of *Wt* was evaluated in a simulation study on the same data set with different window lengths. To understand the algorithm performance in a clinical or pharmacological perspective, we conducted a validation study. We designed 4 pharmacological scenarios and asked 4 independent professionals to compare a traditional method against the smooth algorithm. Data from the simulation and validation studies were then analyzed.

**Results:**

The *Wt* parameter had an impact over the raw data. As we increased the window length, more patient were modified and the number of smoothed patients augmented, although we rarely observed changes of more than 5% of the total data. In the validation study, significant differences were obtained in the performance of the smooth algorithm over the traditional method. These differences were consistent across pharmacological scenarios.

**Conclusions:**

The smooth algorithm is an automated approach that standardizes, simplifies, and improves data processing in drug exposition studies using EHRs. This algorithm can be generalized to almost any pharmacological medication and model the drug exposure to facilitate the detection of treatment switches, discontinuations, and terminations throughout the study period.

## Introduction

The recent rise in the use of electronic health records (EHRs) has had a major impact on epidemiological research. These databases provide a low-cost means of accessing longitudinal data such as demographic, vital signs, administrative, medical and pharmacy claims, clinical, and patient-centered data on large populations for epidemiologic research [1,2].

However, in their current form, EHRs are complex and imperfect data sets that can be enhanced in a dizzying number of often ineffective ways. Although the challenges of working with EHRs in clinical trials have been identified [3-5], more research is needed to develop new and better ways to use them.

From the data mining perspective, addressing these data is particularly challenging as the outcomes can be significantly affected depending on the quality, validity, completeness, and heterogeneity of the available data [6]. Besides technical perspective, researchers have their particular ways of addressing EHR, dealing with EHR complexity, and their decisions have been shown to have a significant impact to the results [7,8]. Approaching these challenges in a heterogeneous and biased way favors the emergence of inconsistencies between similar studies [9].

In studies with EHR-based databases, information on drug exposure is usually obtained from electronic prescription, electronic dispensation, or invoice of drugs. This information is widely used and accepted in clinical research as the availability of longitudinally recorded data allows for a detailed characterization of both the exposure to medication and the outcome of interest, and mining the data contained within EHRs can potentially generate a greater understanding of medication effects in the real world, complementing what we know from randomized control trials [10].

Focusing on pharmacovigilance or pharmacoepidemiology when using EHRs, one of the main objectives is to characterize the therapy in terms of duration [11], discontinuation [12,13], changes [14], and adherence to pharmacological treatments [15]. Although progress has been made in this area for monotherapy [16], when we study treatment exposure in diseases such as hypertension, diabetes, or chronic obstructive pulmonary disease, treatment often switches from monotherapy to combinations of two or more drugs, which significantly increases the challenge of characterizing the treatment. In our experience [17], polytherapy in EHR-based studies creates complex treatment patterns that are challenging to analyze or interpret, can be blinded to researchers, and can be a source of misunderstanding as it is difficult to distinguish whether they are real occurrences or recording errors. To address this, we propose a novel algorithm called *smooth* to obtain the most likely therapy of one or more drugs over time.

## Methods

### Data Sources

We used the Information System for Research in Primary Care (SIDIAP) [18] as our reference EHR platform for the algorithm development. The SIDIAP includes information recorded by health professionals during routine visits at 287 primary health care centers from the Catalan Health Institute (Institut Català de la Salut).

The platform includes information on disease diagnoses (*International Classification for Diseases, 10th Edition*), drug prescriptions and drug invoices in the primary care setting (Anatomical Therapeutic Chemical [ATC] classification system), and clinically relevant parameters (eg, weight, blood pressure, laboratory tests) as well as sociodemographic characteristics. It is also linked to a hospital discharge database for patients admitted to the Catalan Health Institute hospitals (30% of the SIDIAP population). The SIDIAP has pseudonymized records for more than seven million people and is representative of the Catalan population in terms of age, sex, and geographic distribution [19].

For the algorithm development and validation study, we used a subset of patients drawn from all Catalan Health Institute primary care centers. From this population, we also obtained sociodemographic characteristics: sex, age, country of origin, profession, socioeconomic index, smoking habits, alcohol intake, institutionalization in nursing homes, comorbidities, and electronic prescriptions of pharmacological treatments.

**Figure 1:**
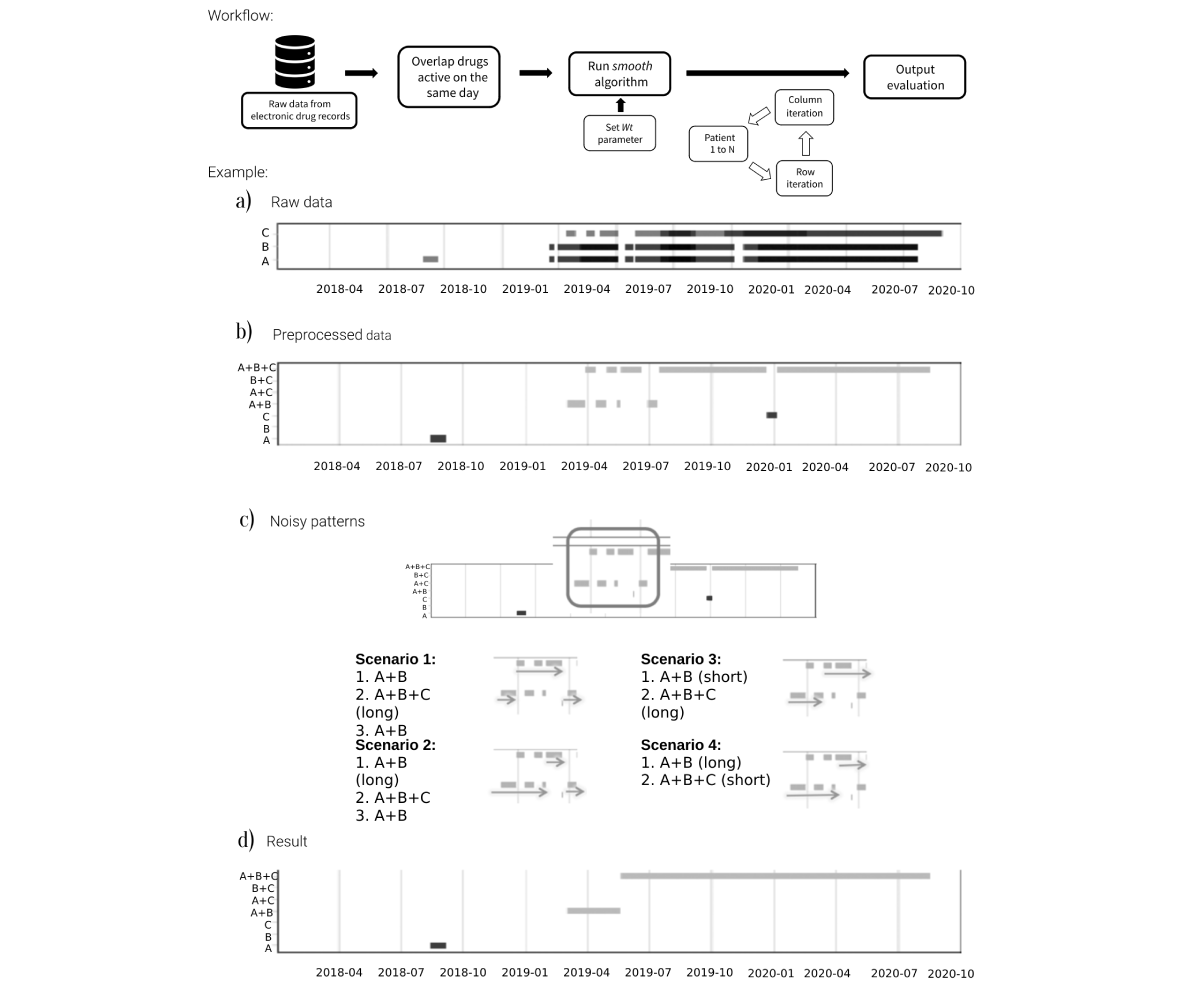
Overview of the smooth algorithm workflow with an illustrated example. (A) Horizontal axis is follow-up time in days, and the vertical axis is the different drugs prescribed. Box length indicates the period in which the prescription is active. (B) Patient profile after combining all active prescriptions in the same day, the first step of data process. (C) Example of complex patterns and 4 ways to overcome them. (D) Result obtained after passing the data through the smooth algorithm.

In SIDIAP, electronical prescriptions and drug invoices are stored in longitudinal format. Each record comprises the pseudonymized patient identifier, ATC code, and prescription or invoice date. The end of the prescription is determined by the health professional, whereas in drug invoice records we only have the month in which the invoice was made, and thus the end of the treatment is usually inferred based on the number of packages collected. Each prescription or invoice is recorded independently of the health problem, and duplicate records or overlaps are common (Figure 1A).

### Ethics Approval

The study protocol was approved by the Research Ethics Committee of Fundació Institut Universitari per a la recerca a l'Atenció Primària de Salut Jordi Gol i Gurina (IDIAPJGol; AR20/029, SIDIAP 386 on June 3, 2020). This is a database research study that has been conducted according to the guidelines of the Declaration of Helsinki (Fortaleza, Brazil 2013) and does not require consent from the people included to participate or for publication. The need for consent was waived by the Research Ethics Committee of IDIAPJGol as it is deemed unnecessary according to European legislation (Regulation [EU] 2016/679).

### Finding the Most Likely Therapy Using the Smooth Algorithm

The process has 2 parts: data mining to look for treatments recorded daily and applying the smooth algorithm to the data (Figure 1).

For the first part, we looked at all drugs of interest that are active on the same day (Figure 1A and Figure 1B). This step simplifies the prescription or invoice records but frequently reveals complex patterns that should be considered before conducting any analysis. Data are processed based on assumptions about those patterns by individual researchers and therefore give nonhomogeneous results. In Figure 1C, we imagined 4 scenarios (but there could be more) to handle the same problem (highlighted area during the first half of 2019). While some researchers may consider that the first therapy lasts until we observe a change in the treatment, others with different backgrounds may decide that a change from double to triple therapy can only be considered if the triple therapy lasts longer than an arbitrary period (eg, 60 days).

The smooth algorithm is inspired by statistical methods using simple moving averages that calculate trends or smooth time series [20]. For EHRs, we changed the concept of moving averages to a moving window from where we choose the most frequent treatment. Thus, by moving the window one day at a time, we identify the most frequent pattern over the study period (Figure 1D).

**Figure 2:**
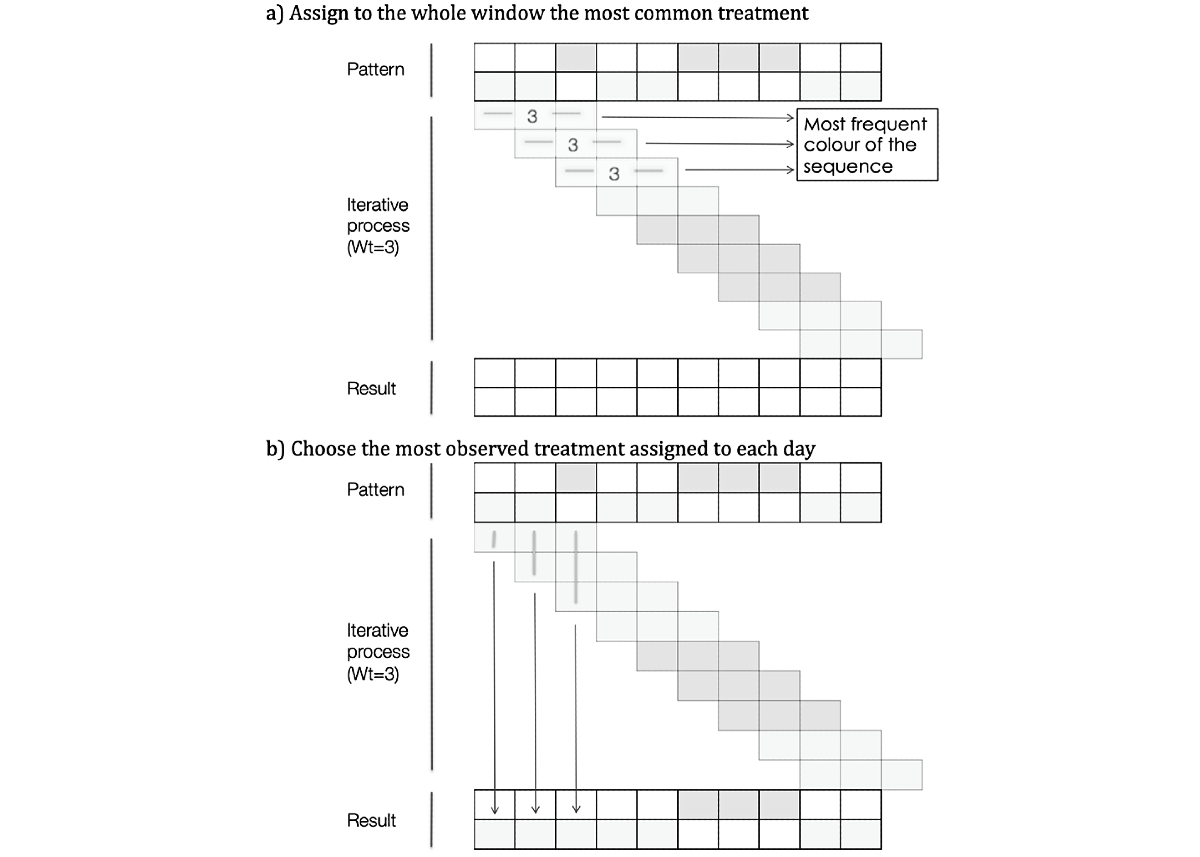
Smooth algorithm in detail.

In Figure 2, we can see a detailed description of the algorithm. It is an iterative process that works as follows:

- Starting from the first prescription or dispensation at day *t_i_*, we opened a window of specific length in days (*Wt*) in which we search for the most frequent treatment
- That treatment was assigned to the whole window unless we had a draw (2 treatments are active for the same number of days), in which case we carried the previous treatment (*t_i-1_*) forward.
- We then shifted the window forward one day at a time, repeating the process until the end of follow-up (Figure 2A).
- After the first iteration, we had up to *Wt* possible treatments (or candidates) for each day
- Finally, we chose the candidate most frequently observed on that day (Figure 2B).

### Window Size

**Figure 3:**
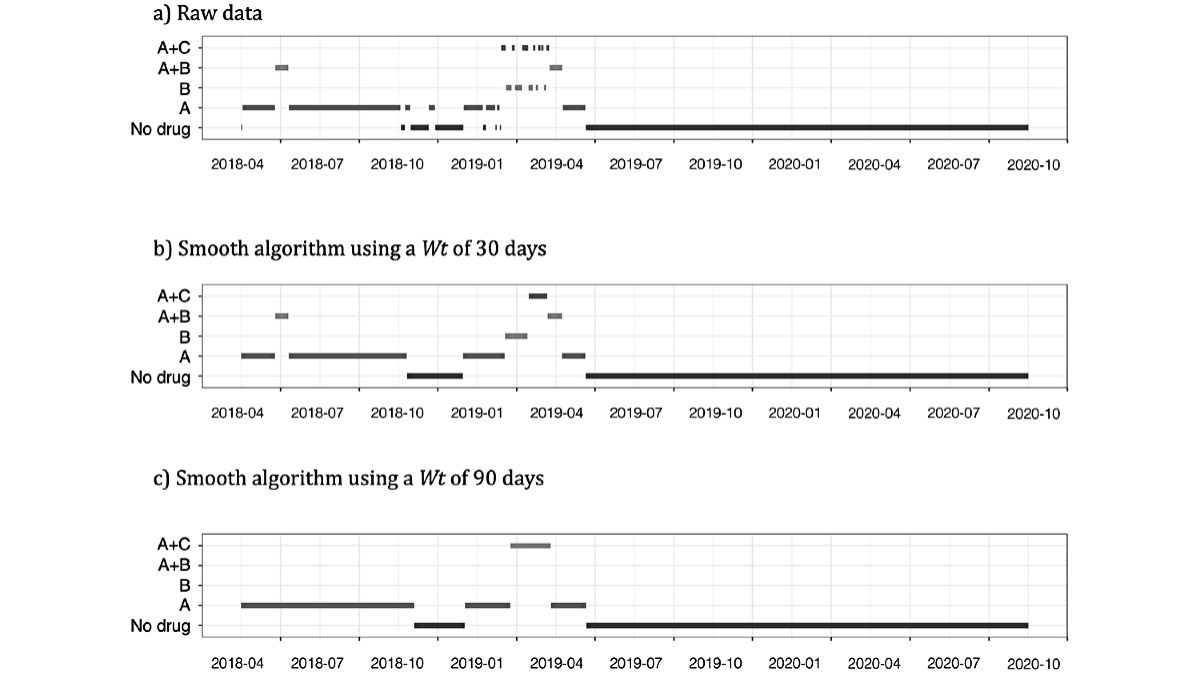
Example of how the outcome changes according to the value of the Wt parameter.

The length of *Wt* is the only parameter that needs to be defined beforehand, and its value can modify the outcome (Figure 3). The length of *Wt* determines the level of smoothing, and the value can go from 1 day to the total days of follow-up. Thus, for *Wt* of 1 day, we are not changing the data while for a *Wt* equal to the number of days of follow-up, we expect to reduce all records to the most frequent treatment. Therefore, small values of the *Wt* parameter will not significantly modify the raw data, whereas increasing the size of the window is expected to have a larger impact on the data.

In a more in-depth analysis, a simulation study was conducted using 7132 patients who were under long-term administration of aspirin, statins, beta-blockers, and angiotensin-converting enzyme (ACE) inhibitors or angiotensin-receptor blockers between 2018 and 2020. The smooth algorithm was run on this data using 6 *Wt* values: 10, 20, 30, 45, 60, and 90 days. For each *Wt*, we counted the number of patients with at least one change in the treatment pattern; out of these, we calculated the percentage of smooth as the ratio of the number of days changed by the algorithm divided by the total number of days with active treatment.

### Validation Study

To understand how the algorithm performs from a clinical or pharmacological perspective, we conducted a validation study. We identified 4 pharmacological scenarios where the algorithm could be needed—combination of 3 or more drugs (platelet aggregation inhibitors, beta-blockers, and ACE inhibitors), treatments likely to discontinue (antidepressants), long-term combination of 2 drugs (insulins and oral antidiabetics), and short-term treatment (systemic antibiotics)—and asked to 4 independent professionals with experience in databases and drug exposure studies to compare a traditional method with the smooth algorithm.

The traditional method is a more intuitive and simple approach, commonly observed in the literature, to address noise and variability in electronic drug records [21,22]. Briefly, it starts with the first treatment observed and accepts a treatment change only if the new one is longer than a certain period of time. This period is generally arbitrary, an assumption done by the researcher based on the characteristics of the drug. For our validation study, we set the period to 60 days except for antibiotics (the short-term treatment), with a period of 15 days. For the smooth algorithm, the *Wt* parameter was set to 60 days in all 4 scenarios.

Before conducting the validation, we prepared a training session with the 4 reviewers consisting in an introduction to the data, drugs of study (including the selected ATC codes), explanation of the algorithms, and discussion of the common criteria to apply during the validation. From their feedback, and after the training, we decided to include all health problems related to the treatment as it may facilitate the evaluation and give more importance to clinical criteria (see validated sample in Multimedia Appendix 1, Figure S1).

Our primary objective was changes to the original data; we analyzed whether the algorithms improved, worsened, or made no changes to the original data. In addition, we asked the reviewers to choose between the traditional method and the smooth algorithm and evaluate its value for detecting treatment switches and**/**or discontinuations.

Each professional reviewed 100 patient records with one-quarter of the records being assigned to all reviewers to analyze consistency across validations. To reduce potential biases, reviewers were blinded and they did not know which method or algorithm generated the results (Table 1).

**Table 1:** Description of drugs and distribution of samples in the validation study.

Treatment pattern of use	Description (ATC^a^ code)	Prescriptions in the data set	Samples analyzed, total (per reviewer)	Samples repeated across reviewers, total (per reviewer)
Short-term drugs	Systemic antibiotics (J01)	10,846,282	80 (20)	40 (10)
Likely to discontinue	Antidepressants (N06a)	3,859,496	80 (20)	40 (10)
Long-term combinations of 2 drugs	Insulins and oral antidiabetics (A10)	22,271,154	120 (30)	60 (15)
Combination of 3 or more drugs	Platelet aggregation inhibitors (B01Ac)	21,253,742	120 (30)	60 (15)

^a^ATC: Anatomical Therapeutic Chemical.

### Statistical Methods

We determined that a sample size of 400 patients would be enough to ensure 80% power assuming a minimum effect size of 0.3062 with two degrees of freedom for a Chi-square test under a significance level of 5%.

Categorical variables were described with relative and absolute frequencies, and results from numerical variables were reported using means and standard deviations. For the validation study, we used the Chi-square test to evaluate differences between the traditional method and smooth algorithm on performance compared with the raw data. The algorithm was programmed in R (version 4.1.0, R Foundation for Statistical Computing), and all analyses were performed in R.

## Results

### Impact of *Wt* Parameter and Simulation Study

In Figure 3, we show how the results changed according to the *Wt* value. In the example, for the raw data we note that during the first period of follow-up, the main treatment was a monotherapy followed by a complex pattern during the first 6-month semester of 2019 (Figure 3A). To reduce noise in the pattern, we applied the smooth algorithm using *Wt* values of 30 and 90 days. With a *Wt* of 30 days (Figure 3B), we retained the combination of 2 drugs during the early stages of follow-up; during months with more changes, patient moved from monotherapy to a combination of 2 drugs (A+C and A+B) prior to switching to the A monotherapy. In contrast, with a *Wt* of 90 days (Figure 3C), the entire treatment pattern was simplified. During the first year of follow-up, we observed a monotherapy; during the most complex pattern, the algorithm smoothed the changes to a single combination of A+C before returning to a monotherapy.

**Figure 4:**
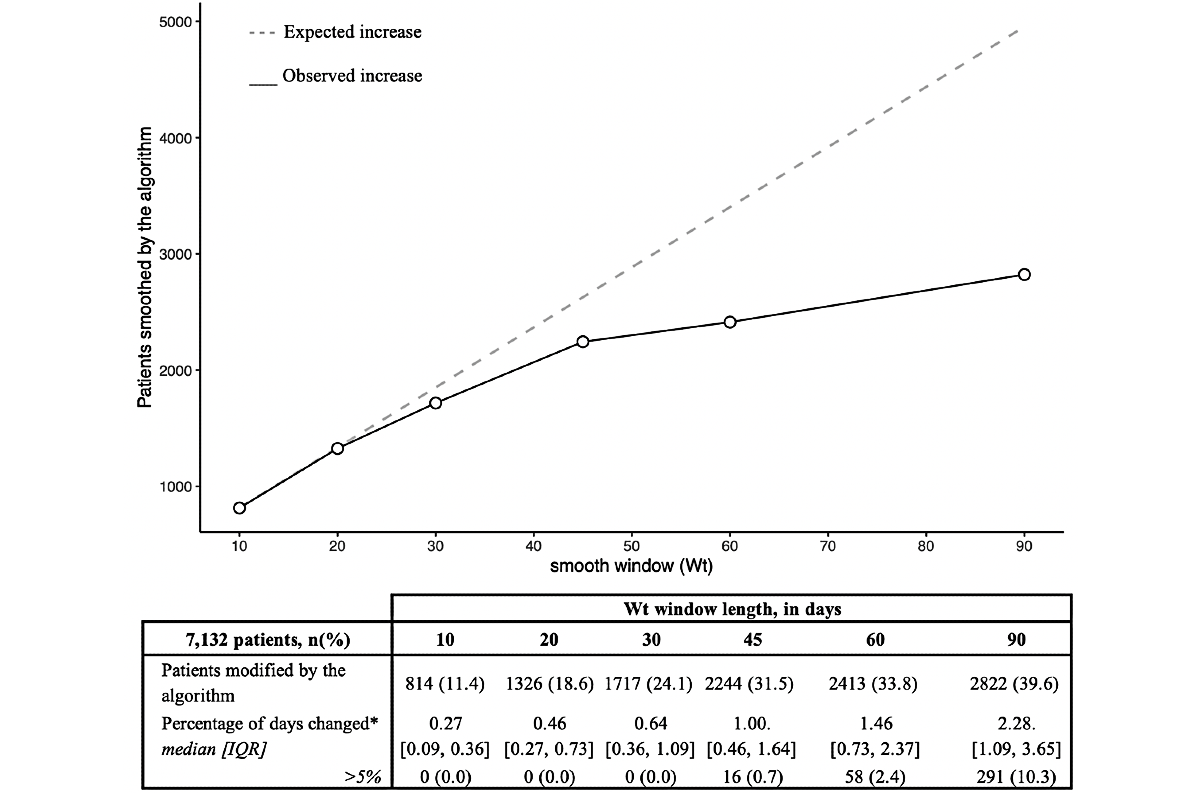
Statistics of the simulation study using 6 Wt values on 7132 patients under treatment for cardiovascular disease between 2018 and 2020. *100 x Number of days changed / Total days under prescription.

Results of the simulation study are represented in Figure 4. With a *Wt* of 10 days, 11.4% (814/7132) had their treatment pattern changed, while with a 90-day window, 39.6% (2822/7132) had their treatment pattern modified; 31.5% (2244/7132), 33.8% (2413/7132), and 39.6% (2822/7132) of patients with Wt values of 45, 60, and 90 days, respectively, saw at least 1 change. Thus, the effect of *Wt* was not linear as the expected progression of the number of patients being smoothed was different than the results from the simulation.

As a relative measure, we reported the percentage of days changed, ranging from 0.27 (IQR 0.09, 0.36) to 2.28 (IQR 1.09, 3.65). Thus, for each of the *Wt* values 10, 20, 30, 45, 60, and 90 days in a 1000-day period of follow-up, the algorithm modified 2.7, 4.6, 6.4, 10, 14.6, and 22.8 days, respectively. At windows from 10 to 30 days, the percentage of days changed always remain below the 5%, but as we increased the *Wt* to 45, 60, and 90 days, we started to observe patients with more than 5% of the data smoothed.

### Validation Study

Multimedia Appendix 2 includes the results of the validation study. For the 400 samples, the smooth algorithm improved the raw data for 56.8% (227/400) of individuals, while 42.5% (170/400) benefited from using the traditional method. In 39% (156/400) of the samples, the outcome provided by each algorithm did not change the patterns, and 4.2% (17/400) of cases reported worsening after being processed by the smooth algorithm. The traditional method resulted in a worse outcome for 18.5% (74/400) of the samples, and the observed differences between algorithms were statistically significative (*P*<.001).

Significant differences were also observed between algorithms stratified by scenario. With a combination of 3 or more drugs (platelet aggregation inhibitors, beta-blocker, and ACE inhibitors), drugs likely to be discontinued (antidepressants), and the combination of 2 or more long-term drugs (insulins and antidiabetics), the smooth algorithm improved 69.2% (83/120), 61.3% (49/80), and 60.0% (72/120) of samples, respectively. In short-term treatment (systemic antibiotics), 90.0% (72/80) of samples did not show changes and 28.7% (23/80) were improved by the smooth algorithm.

As for the samples validated by the 4 professionals (Table 1), they decided in 88.0% (44/50) of patients to choose the smooth algorithm over the traditional method (Multimedia Appendix 2 and Multimedia Appendix 1, Figure S2). It was in the short-term treatment scenario where we observed less of a consensus, 70% (7/10), whereas for the rest of scenarios the 4 reviewers agreed in 93.3% (14/15), 100% (10/10), and 86.7% (13/15) of the samples, respectively.

The smooth algorithm performed better than the traditional method in detecting discontinuations (350/366, 95.6%) and treatment switches (138/230, 60.0%; see Multimedia Appendix 1, Table S1).

## Discussion

### Principal Findings

Since the use of EHR databases began in pharmacoepidemiologic studies, researchers have been trying to establish algorithms to model drug exposure [23]. This becomes even more challenging when trying to assess drug exposures with multiple pharmacologic treatments, which happens quite often in older people, as they are prescribed with up to 5 drugs simultaneously [24]. Thus, we have developed an automatic algorithm to model drug exposure through EHRs, which standardizes the data mining process to obtain more consistent and replicable results across studies.

The algorithm is inspired by time series forecasting methods and requires a parameter to be set beforehand. This is commonly observed in similar statistical methods such as autoregressive models or moving averages [25], and it is known that the value of the parameter can modify the outcome significantly [26-28]. The simulation study shows the impact of the *Wt* value. Small values hardly change the original data, but as the parameter value increases, the raw data can be affected to the point of losing clinical relevance. In the worst-case scenario, we observed changes in up to 40% of the patients, with 75% of those having at least 1% of the records smoothed.

Interestingly, the simulation shows that at a certain *Wt* value, the number of individuals modified reaches a plateau. The data changed by the algorithm are less than expected, particularly when *Wt* is greater than 30 days, suggesting that independently of the parameter, some patients will never be changed by the algorithm.

In the validation study, we observed that most times our algorithm improved the data patterns. It was designed to improve polypharmacy exposure assessment, and we were interested in the results for combinations of 3 or more drugs. In this scenario, both the traditional and smooth approaches demonstrated usefulness, and the percentages of improved samples were similar, although the smooth algorithm performed significantly better. These differences were also observed in the other scenarios, and we believe that the smooth algorithm not only improves the treatment pattern but also does not make it worse. In addition, the performance was not affected by the window length, since for antibiotics and antidepressants (short- and long-term drug use, respectively) the smooth algorithm performed well using the same *Wt* window.

The traditional method proved to be a good approach, and similar versions are being used in other studies [21,22,29], but it differs according to drug or study characteristics and so is less generalizable. In fact, it has not worked well for systemic antibiotics even though we specifically changed it to fit for its characteristics. Overall, the validations for the smooth algorithm were consistent between scenarios and reviewers.

### Potential Uses and Strengths

We believe that the smooth algorithm has significant potential to assess exposure for treatment combinations, especially in chronic treatments, since it allows us to have a time sequence of exposure to the treatment. This sequence allows us to better model the drug exposition and detect discontinuations, switches, and periods of interaction with other drugs of short duration. In addition, it can be of great help in estimating adherence to a combination treatment [30]. With the smooth algorithm, we can easily calculate the exposure time using only electronic prescriptions without the need to know the dosage posology.

From a clinical point of view, the smooth algorithm has great advantage when estimating polypharmacy adherence [31]. Patients affected by chronic conditions in need of polypharmacy may have differing levels of adherence to individual medications within their regimen, and this could lead to varying health outcomes and misleading results if the methodological approach assumes as equivalent adherence to all medications. These patients also face other acute conditions requiring the addition of drugs or modification of doses while maintaining their actual medication regimen.

Another research area in which our algorithm could be of utility is the study of adverse events due to lack of effectivity (antibiotics or hypertension treatments) and drug-drug interactions (anticoagulants and nonsteroidal anti-inflammatories combined for short time periods).

Although we cannot ever know a patient’s true adherence, the smooth algorithm is an automated way to analyze EHR data offering methodological consistency across studies. In several studies, assumptions were made prior to the data processing, and this may have impacted the results of the analyses by introducing bias on the final results [32]. Algorithms like smooth can help standardize these assumptions and minimize inconsistencies between studies with similar databases.

### Limitations

Due to the nature of the algorithm eliminating complex patterns, we may lose relevant exposures of a short period of time, and smooth is not recommended for all scenarios. Similarly, smooth may not work well in long-acting drugs.

We were not able to capture the posology with the SIDIAP database, so we could not estimate the length of treatment through the dose prescribed. The treatment doses can change throughout the year (decreased use of diuretics during summertime, when traveling, etc).

From a technical perspective, using the smooth algorithm is a time-consuming process. To run the algorithm on big data sets like EHRs, good information technology is needed. The time needed to complete the process may vary depending on the number of patients and follow-up time.

In addition, before running the algorithm, we must set a parameter, *Wt*, that allows us to choose between precision and simplicity. Setting this parameter is not straightforward, and it is important to understand the effect on the outcome to use a good value. This is an inherent limitation of the algorithm and, as a guide, we recommend setting the *Wt* value within the range of 30 to 60 days to reduce complex patterns without compromising relevant information. Moreover, the *Wt* can be changed so the algorithm can be used in short (antibiotics) and long-term (antidepressants) treatments as well as in drug combinations for chronic conditions such as diabetes and hypertension.

Another limitation of the study is that the validation was done with the traditional method instead of other algorithms as a comparator. Moreover, in our experience, we commonly see the traditional method being used with some differences or criteria according to the framework or objectives of the study. For example, in projects with the European Medicines Agency, we have never seen a method or an automatic approach to deal with drug exposure other than the one we call traditional [33].

### Conclusion

The smooth algorithm is an automated approach to estimate the most likely drug exposure pattern. We proved that it standardizes, simplifies, and improves the data processing steps before performing the study analysis; can model the drug exposure to detect cotreatment, switches, discontinuations, and treatment terminations; and facilitates adherence calculations throughout the study period. In future pharmacoepidemiological studies, we aim to further validate the algorithm and analyze the impact the algorithm can have on the main results.

## Appendix

Multimedia Appendix 1 Supplementary material.

Multimedia Appendix 2 Results of the validation study.

## Abbreviations

ACE: angiotensin-converting enzyme
ATC: Anatomical Therapeutic Chemical
EHR: electronic health record
IDIAPJGol: Fundació Institut Universitari per a la recerca a l'Atenció Primària de Salut Jordi Gol i Gurina
SIDIAP: Information System for Research in Primary Care

## References

[1] Casey JA, Schwartz BS, Stewart WF, Adler NE (2016). Using electronic health records for population health research: a review of methods and applications. Annu Rev Public Health.

[2] Rogers JR, Lee J, Zhou Z, Cheung YK, Hripcsak G, Weng C (2021). Contemporary use of real-world data for clinical trial conduct in the United States: a scoping review. J Am Med Inform Assoc.

[3] Cowie MR, Blomster JI, Curtis LH, Duclaux S, Ford I, Fritz F, Goldman S, Janmohamed S, Kreuzer J, Leenay M, Michel A, Ong S, Pell JP, Southworth MR, Stough WG, Thoenes M, Zannad F, Zalewski A (2017). Electronic health records to facilitate clinical research. Clin Res Cardiol.

[4] Lu Z (2009). Information technology in pharmacovigilance: benefits, challenges, and future directions from industry perspectives. Drug Healthc Patient Saf.

[5] Menachemi N, Collum TH (2011). Benefits and drawbacks of electronic health record systems. Risk Manag Healthc Policy.

[6] Weiskopf NG, Weng C (2013). Methods and dimensions of electronic health record data quality assessment: enabling reuse for clinical research. J Am Med Inform Assoc.

[7] Salas M, Lopes LC, Godman B, Truter I, Hartzema AG, Wettermark B, Fadare J, Burger JR, Appenteng K, Donneyong M, Arias A, Ankrah D, Ogunleye OO, Lubbe M, Horne L, Bernet J, Gómez-Galicia DL, Del Carmen Garcia Estrada M, Oluka MN, Massele A, Alesso L, Herrera Comoglio R, da Costa Lima E, Vilaseca C, Bergman U (2020). Challenges facing drug utilization research in the Latin American region. Pharmacoepidemiol Drug Saf.

[8] Rudin RS, Friedberg MW, Shekelle P, Shah N, Bates DW (2020). Getting value from electronic health records: research needed to improve practice. Ann Intern Med.

[9] Wang SV, Schneeweiss S, Berger ML, Brown J, de Vries F, Douglas I, Gagne JJ, Gini R, Klungel O, Mullins CD, Nguyen MD, Rassen JA, Smeeth L, Sturkenboom M, Joint ISPE-ISPOR Special Task Force on Real World Evidence in Health Care Decision Making (2017). Reporting to improve reproducibility and facilitate validity assessment for healthcare database studies V1.0. Pharmacoepidemiol Drug Saf.

[10] Yao L, Zhang Y, Li Y, Sanseau P, Agarwal P (2011). Electronic health records: implications for drug discovery. Drug Discov Today.

[11] Støvring H, Pottegård A, Hallas J (2016). Determining prescription durations based on the parametric waiting time distribution. Pharmacoepidemiol Drug Saf.

[12] Lyu H, Zhao S, Yoshida K, Tedeschi S, Xu C, Nigwekar S, Leder B, Solomon D (2020). Delayed denosumab injections and bone mineral density response: an electronic health record-based study. J Clin Endocrinol Metab.

[13] Mascarenhas J, Mehra M, He J, Potluri R, Loefgren C (2020). Patient characteristics and outcomes after ruxolitinib discontinuation in patients with myelofibrosis. J Med Econ.

[14] Sharman J, Kabadi SM, Clark J, Andorsky D (2021). Treatment patterns and outcomes among mantle cell lymphoma patients treated with ibrutinib in the United States: a retrospective electronic medical record database and chart review study. Br J Haematol.

[15] Li X, Cole SR, Westreich D, Brookhart MA (2018). Primary non-adherence and the new-user design. Pharmacoepidemiol Drug Saf.

[16] Suchard MA, Schuemie MJ, Krumholz HM, You SC, Chen R, Pratt N, Reich CG, Duke J, Madigan D, Hripcsak G, Ryan PB (2019). Comprehensive comparative effectiveness and safety of first-line antihypertensive drug classes: a systematic, multinational, large-scale analysis. Lancet.

[17] Giner-Soriano M, Sotorra Figuerola G, Cortés J, Pera Pujadas H, Garcia-Sangenis A, Morros R (2018). Impact of medication adherence on mortality and cardiovascular morbidity: protocol for a population-based cohort study. JMIR Res Protoc.

[18] Bolíbar B, Fina AF, Morros R, Garcia-Gil MDM, Hermosilla E, Ramos R, Rosell M, Rodríguez J, Medina M, Calero S, Prieto-Alhambra D, Grupo S (2012). [SIDIAP database: electronic clinical records in primary care as a source of information for epidemiologic research]. Med Clin (Barc).

[19] García-Gil M, Hermosilla E, Prieto-Alhambra D, Fina F, Rosell M, Ramos R, Rodriguez J, Williams T, Van Staa T, Bolíbar B (2011). Construction and validation of a scoring system for the selection of high-quality data in a Spanish population primary care database (SIDIAP). Inform Prim Care.

[20] Watson GS (1964). Smooth regression analysis. Sankhyā Indian J Stat.

[21] Xu H, Aldrich M, Chen Q, Liu H, Peterson N, Dai Q, Levy M, Shah A, Han X, Ruan X, Jiang M, Li Y, Julien J, Warner J, Friedman C, Roden D, Denny J (2015). Validating drug repurposing signals using electronic health records: a case study of metformin associated with reduced cancer mortality. J Am Med Inform Assoc.

[22] Hughey J, Colby J (2019). Discovering cross-reactivity in urine drug screening immunoassays through large-scale analysis of electronic health records. Clin Chem.

[23] Andrade SE, Kahler KH, Frech F, Chan KA (2006). Methods for evaluation of medication adherence and persistence using automated databases. Pharmacoepidemiol Drug Saf.

[24] Pazan F, Wehling M (2021). Polypharmacy in older adults: a narrative review of definitions, epidemiology and consequences. Eur Geriatr Med.

[25] Hannan EJ, Rissanen J (1983). Recursive estimation of mixed autoregressive-moving average order. Biometrika.

[26] Durbin J (1959). Efficient estimation of parameters in moving-average models. Biometrika.

[27] Sandgren N, Stoica P, Babu P (2012). On moving average parameter estimation. Eur Signal Process Conf.

[28] Żebrowska M, Dzwiniel P, Waleszczyk WJ (2020). Removal of the sinusoidal transorbital alternating current stimulation artifact from simultaneous EEG recordings: effects of simple moving average parameters. Front Neurosci.

[29] van Staa T, Abenhaim L, Leufkens H (1994). A study of the effects of exposure misclassification due to the time-window design in pharmacoepidemiologic studies. J Clin Epidemiol.

[30] Vlacho B, Mata-Cases M, Mundet-Tudurí X, Vallès-Callol J, Real J, Farre M, Cos X, Khunti K, Mauricio D, Franch-Nadal J (2021). Analysis of the adherence and safety of second oral glucose-lowering therapy in routine practice from the mediterranean area: a retrospective cohort study. Front Endocrinol (Lausanne).

[31] Franklin JM, Gopalakrishnan C, Krumme AA, Singh K, Rogers JR, Kimura J, McKay C, McElwee NE, Choudhry NK (2018). The relative benefits of claims and electronic health record data for predicting medication adherence trajectory. Am Heart J.

[32] Pye SR, Sheppard T, Joseph RM, Lunt M, Girard N, Haas JS, Bates DW, Buckeridge DL, van Staa TP, Tamblyn R, Dixon WG (2018). Assumptions made when preparing drug exposure data for analysis have an impact on results: an unreported step in pharmacoepidemiology studies. Pharmacoepidemiol Drug Saf.

[33] Cid-Ruzafa J, Lacy BE, Schultze A, Duong M, Lu Y, Raluy-Callado M, Donaldson R, Weissman D, Gómez-Lumbreras A, Ouchi D, Giner-Soriano M, Morros R, Ukah A, Pohl D (2022). Linaclotide utilization and potential for off-label use and misuse in three European countries. Therap Adv Gastroenterol.

