# Supplementary material for: Automatic Estimation of the Most Likely Drug Combination in Electronic Health Records Using the Smooth Algorithm: Development and Validation Study
Source: JMIR Med Inform. 2022 Nov 15;10(11):e37976. doi: 10.2196/37976 (PMC9709675; doi:10.2196/37976)
Supplement: Multimedia Appendix 1 [file medinform_v10i11e37976_app1.docx]

**Multimedia Appendix 1. Supplementary materials.**

**Figure S1. Example of a validated sample under antibiotics (short-term treatment)**


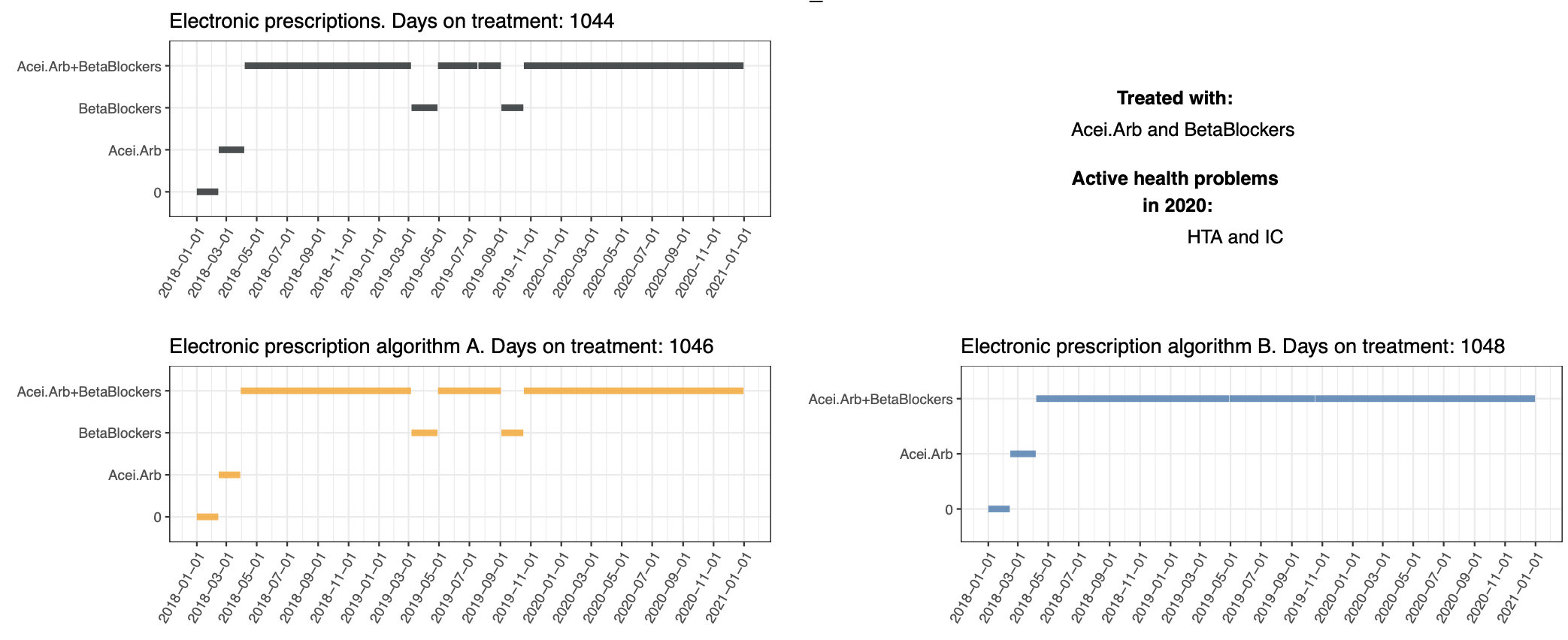


**Figure S2. Concordance between reviewers in the validation study**

**Times they choosed the smooth algorithm againts raw data**
