# Supplementary material for: Automatic Estimation of the Most Likely Drug Combination in Electronic Health Records Using the Smooth Algorithm: Development and Validation Study
Source: JMIR Med Inform. 2022 Nov 15;10(11):e37976. doi: 10.2196/37976 (PMC9709675; doi:10.2196/37976)
Supplement: Multimedia Appendix 2 [file medinform_v10i11e37976_app2.docx]

**Multimedia Appendix 2. Results of the validation study. Overall and by type of drug.**

|  | **All treatments** | | | **Combination of three or more drugs (Antiplatelets, beta blocking and ace inhibitors)** | | | **Likely to discontinue (Antidepressants)** | | | **Long-term two drug combination. (Insulines and oral antidiabetics)** | | | **Short-term treatment (Systemic antibiotics)** | | |
| --- | --- | --- | --- | --- | --- | --- | --- | --- | --- | --- | --- | --- | --- | --- | --- |
| **Each algorithm compared to raw data (n, %)** | | | | | | | | | | | | | | | |
|  | **Traditional** | **Smooth** | ***P***^a^ | **Traditional** | **Smooth** | ***P***^a^ | **Traditional** | **Smooth** | ***P***^a^ | **Traditional** | **Smooth** | ***P***^a^ | **Traditional** | **Smooth** | ***P***^a^ |
| n | 400 | 400 | <.001 | 120 | 120 | .006 | 80 | 80 | <.001 | 120 | 120 | .015 | 80 | 80 | <.001 |
| Equal or improved | 326 (81.5) | 383 (95.8) |  | 106 (88.3) | 118 (98.3) |  | 63 (91.2) | 79 (98.8) |  | 100 (83.3) | 114 (95.0) |  | 47 (58.7) | 72 (90.0) |  |
| *Improved* | 170 (42.5) | 227 (56.8) |  | 79 (65.8) | 83 (69.2) |  | 14 (17.5) | 49 (61.3) |  | 63 (52.5) | 72 (60.0) |  | 14 (17.5) | 23 (28.7) |  |
| **Concordance between reviewers (n, %)** | | | | | | | | | | | | | | | |
| Patients reviewed by all 4 specialists | 50 | | | 15 | | | 10 | | | 15 | | | 10 | | |
| *Agreed* | 44 (88.0) | | | 14 (93.3) | | | 10 (100.0) | | | 13 (86.7) | | | 7 (70.0) | | |

^a^ Chi-square test for the performance comparison between tradition method and smooth algorithm against raw data
